# The distinct and overlapping brain networks supporting semantic and spatial constructive scene processing

**DOI:** 10.1016/j.neuropsychologia.2021.107912

**Published:** 2021-07-30

**Authors:** Cornelia McCormick, Eleanor A. Maguire

**Affiliations:** a Wellcome Centre for Human Neuroimaging, UCL Queen Square Institute of Neurology, University College London, London, WC1N 3AR, UK; b Department of Neurodegenerative Diseases and Geriatric Psychiatry, University Hospital Bonn, Bonn, Germany

**Keywords:** Functional and effective connectivity, Hippocampus, Impossible scenes, Scene construction, Semantic, Ventromedial prefrontal cortex

## Abstract

Scene imagery features prominently when we recall autobiographical memories, imagine the future and navigate around in the world. Consequently, in this study we sought to better understand how scene representations are supported by the brain. Processing scenes involves a variety of cognitive processes that in the real world are highly interactive. Here, however, our goal was to separate semantic and spatial constructive scene processes in order to identify the brain areas that were distinct to each process, those they had in common, and the connectivity between regions. To this end, participants searched for either semantic or spatial constructive impossibilities in scenes during functional MRI. We focussed our analyses on only those scenes that were possible, thus removing any error detection that would evoke reactions such as surprise or novelty. Importantly, we also counterbalanced possible scenes across participants, enabling us to examine brain activity and connectivity for the same possible scene images under two different conditions. We found that participants adopted different cognitive strategies, which were reflected in distinct oculomotor behaviour, for each condition. These were in turn associated with increased engagement of lateral temporal and parietal cortices for semantic scene processing, the hippocampus for spatial constructive scene processing, and increased activation of the ventromedial prefrontal cortex (vmPFC) that was common to both. Connectivity analyses showed that the vmPFC switched between semantic and spatial constructive brain networks depending on the task at hand. These findings further highlight the well-known semantic functions of lateral temporal areas, while providing additional support for the previously-asserted contribution of the hippocampus to scene construction, and recent suggestions that the vmPFC may play a key role in orchestrating scene processing.

## Highlights

• Semantic and spatial constructive scene processing rely on distinct brain regions.
• Temporal cortex supports semantic and hippocampus constructive scene processing.
• vmPFC supports both types of scene processing but with different time courses.
• vmPFC changes connectivity based on the need for semantic or constructive processes.

## 1 Introduction

During our lives we accrue knowledge and build expectations about the appearance of the visual world. One way we can learn about how this critical knowledge is supported by the brain is by examining what happens when violations occur. In an early positron emission tomography study, Schacter et al. (1995) had participants study drawings of novel single objects and they had to judge whether they were structurally possible or impossible. Increased blood flow in inferior temporal regions was associated with object decisions about possible but not impossible objects. Using similar stimuli, Lee and Rudebeck (2010) examined how the objects were processed by two patients, one with bilateral hippocampal damage and the other with similar lesions that extended into perirhinal cortex. Only the patient with the perirhinal damage was impaired on the task, with the deficit being a failure to identify impossible objects while performance on possible objects was not significantly different to control participants.

Given that scenes more closely mirror our experience of the world, Douglas et al. (2017) used functional MRI (fMRI) to move beyond single objects to examine brain responses to spatially possible and impossible Escher-like scenes. They found that increased hippocampal activity was associated with detecting spatially impossible scenes, while posterior parahippocampal cortex was more engaged by spatially possible scenes. McCormick et al. (2017a) also examined possible and impossible scenes, but drew an additional distinction. They noted that spatial but also semantic elements are important features when processing the visual world. In reality these components likely interact but nevertheless might preferentially depend upon different brain structures. Consequently, they examined scenes that were spatially possible or impossible and scenes that were semantically possible or impossible.

The impossible stimuli were either scenes that contained elements that were constructed such that the physical structure was impossible, for example, an Escher-like staircase, or had semantic content that was unfeasible for that scene, for example an elephant with butterflies for ears. They found that patients with focal bilateral hippocampal damage performed comparably to control participants when deciding whether scenes were semantically possible or impossible, but were selectively impaired at judging if scenes were spatial constructively possible or impossible (see Aly et al., 2013; Ruiz et al., 2020; McCormick et al., 2021, for related results). Post-task debriefing indicated that control participants constructed flexible mental representations of the scenes in order to make the spatial constructive judgements, whereas the patients were more constrained and typically focused on specific fragments of the scenes, with little indication of having constructed internal scene models. McCormick et al. (2017a) suggested that these results support the view that one function of the hippocampus is to construct internal representations of spatially coherent scenes (Hassabis and Maguire, 2007; Maguire and Mullally, 2013).

Aligning with these findings, patients with hippocampal damage are able to provide appropriate semantic content associated with scenes (Hassabis et al., 2007a), suggesting that it is not necessary to construct an internal model of a spatially coherent scene for this purpose. This is in contrast to patients with semantic dementia who have atrophy of lateral temporal cortex and who struggle with semantic processing (Warrington, 1975; Snowden et al., 1989; Hodges et al., 1992, 1995; Mummery et al., 2000; Jefferies and Lambon Ralph, 2006; Lambon Ralph et al., 2007; Noppeney et al., 2007). Indeed, neuroimaging studies have shown that lateral temporal, and lateral and medial parietal cortices are more engaged by semantic scene processing (Chadwick et al., 2016; McAndrews et al., 2016; Silveira et al., 2012). Overall, extant neuropsychological and neuroimaging studies have shown that the hippocampus is not engaged by (Schacter et al., 1995), nor does hippocampal damage alone affect (Lee and Rudeback, 2010), the detection of spatial constructive impossibilities in single objects. By contrast, the hippocampus is involved in, and required for, the identification of spatial constructive violations in scenes (Douglas et al., 2017; McCormick et al., 2017a; but see Urgolites et al., 2019), but not for semantic violations in scenes, which may be supported by regions such as lateral temporal cortex.

There is, however, another brain area that is pertinent to consider in this context – the ventromedial prefrontal cortex (vmPFC). The vmPFC has strong anatomical and functional connections with the hippocampus and lateral temporal cortex (Andrews-Hanna et al., 2010; Catani et al., 2012). Together, these structures are often co-activated during fMRI studies involving scene processing (Zeidman et al., 2015; McCormick et al., 2021), and tasks that are known to involve scene imagery (Clark et al., 2020) such as autobiographical memory recall, future thinking and spatial navigation (Svoboda et al., 2006; Spreng et al., 2009).

The vmPFC is thought to instantiate superordinate knowledge structures that reflect abstracted commonalities across multiple experiences, known as schemas (Ghosh and Gilboa, 2014; Gilboa and Marlatte, 2017). It has been suggested that the vmPFC activates context-relevant schemas that are used to guide autobiographical memory recall and the storage of new information, and that it facilitates predictions about how we expect to world to be (Ghosh et al., 2014; Gilboa et al., 2006). Indeed, patients with damage to the vmPFC exhibit deficits that suggest aberrant schema re-activation (Ciaramelli et al., 2006; Gilboa et al., 2006; Ghosh et al., 2014).

Given this role in supporting schema and knowledge of prototypical scenes, the prediction might be that the vmPFC would be engaged by both the semantic and spatial constructive possible/impossible scenes tasks. To test this idea, De Luca et al. (2019) examined patients with vmPFC lesions on McCormick et al.’s (2017a) possible/impossible scenes task. They found no differences between the patients and control participants in detecting either semantic or spatial constructive violations. However, it is notable that vmPFC patients are able to construct scene imagery when the cues provided are specific (Kurczek et al., 2015). It may therefore have been that the demands of the possible/impossible scene task acted as specific and detailed cues and this, along with their intact hippocampi and lateral temporal cortices, helped the vmPFC patients to circumvent any performance issues. Consequently, it remains unknown whether the vmPFC would be engaged during the semantic and spatial constructive possible/impossible scenes tasks.

Another issue concerns functional and effective connectivity between brain areas. For example, recent magnetoencephalography (MEG) studies have shown that the vmPFC drives activity in the hippocampus and lateral temporal cortex during the construction of scene imagery (Barry et al., 2019; Monk et al., 2020, 2021) and also during autobiographical memory recall (McCormick et al., 2020). This has prompted the suggestion that the vmPFC may play a key role in orchestrating scene processing perhaps because of its role in supporting schema (McCormick et al., 2018; Ciaramelli et al., 2019). Whether functional and effective connectivity between the vmPFC and other regions changes dependent upon specific aspects of scene processing is unclear. It may be that vmPFC-lateral temporal cortex connectivity increases during semantic aspects of scene processing, and vmPFC-hippocampal connectivity increases during spatial constructive scene processing.

One feature of all the possible/impossible tasks reported to date is that they involved participants spotting impossibilities which relied on error detection processes that evoked reactions such as surprise, novelty or ‘a-ha’ responses. By contrast, in the current study, healthy participants searched mostly possible scenes, and these were the focus of the fMRI analyses. We included a smaller number of impossible scenes to encourage engagement with the task. Importantly, we counterbalanced the possible scenes across participants, so that half of the participants looked for semantic and the other half of participants looked for spatial constructive impossibilities for a particular possible scene. In this way we could examine the brain states associated with either semantic or spatial constructive processing involving the same (possible) scenes. In addition, we recorded eye-tracking data during scanning. While there is increasing interest in relating oculomotor behaviour to hippocampal engagement, it is unclear whether semantic or spatial constructive aspects of scene processing would result in differences in fixation counts, fixation duration, saccade counts and saccade amplitude. Consequently, as an auxiliary, exploratory arm of the study, we examined oculomotor behaviour in this context. We also performed a post-scan surprise memory task to examine the potential effects of incidental encoding, and asked participants about the cognitive strategies they used to perform the tasks.

Based on the extant literature summarised above, we expected that lateral temporal areas would be particularly engaged during the semantic condition, and the hippocampus specifically during the spatial constructive condition. We further predicted that the vmPFC would be involved in both tasks, and that this would also be expressed in functional and effective connectivity findings, with the vmPFC increasing connectivity with lateral temporal regions during semantic processing and the hippocampus during spatial constructive scene processing.

## 2 Materials and methods

### 2.1 Participants

Twenty five healthy, right-handed participants (10 males, age range 19–35 years, mean age 24.8, SD 4.7; mean years of education 17.2, SD 2.8) were tested in the study. Due to the pictorial nature of the paradigm, we excluded individuals engaging (as professionals, students or hobbyists) in any intensive art-related activities, including artists, architects and designers. All participants gave informed written consent in accordance with the University College London research ethics committee.

### 2.2 Stimuli

**Fig. 1:**
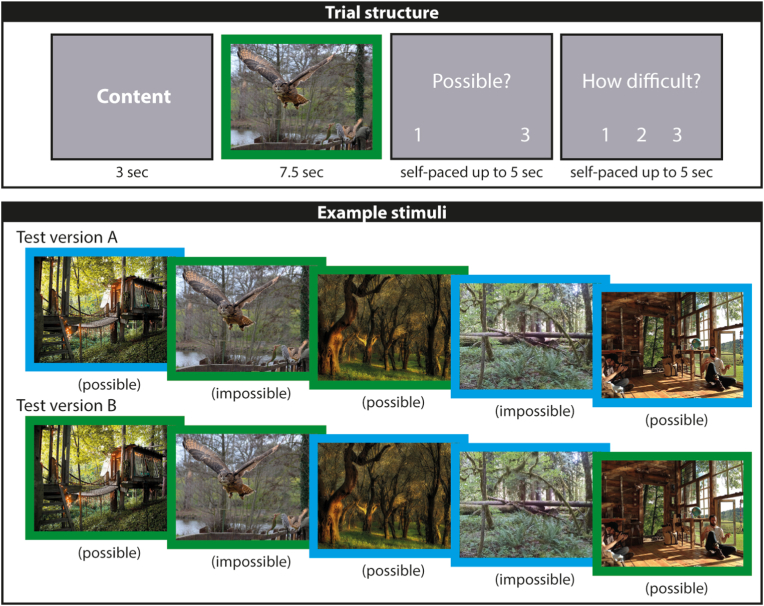
Trial structure and example stimuli. The upper panel shows an example trial. For 3 s, participants saw a cue (either “content” indicating they should search for semantic impossibility on this trial, or “structure” directing them to search for spatial constructive impossibility), followed by the presentation of a scene image for 7.5sec. The background of the image also indicated which impossibility type they should look out for (green = semantic, blue = structure; the scene here is impossible). Participants then indicated their decision (possible, 1 = yes or 3 = no) and made a difficulty rating (1 = not at all, 2 = somewhat, and 3 = very) in a self-paced manner up to a maximum of 5 s. The lower panel shows some example stimuli. While participants who did test version A were cued to search for spatial constructive impossibility in a particular possible scene, participants who did test version B were cued to search for semantic impossibility in the same scene. This procedure allowed us to counterbalance all possible scene images across participants. Impossible catch trials were identical regardless of group membership. Here, for the reader's information, we indicate in brackets whether a scene was possible or impossible; participants never saw such labels. Of note, the semantic impossible scene shown here depicts a forest with a flying seahorse in the lower right corner. The spatial constructive impossible scene above shows a forest where the horizontal tree appears to be in front of and at the back of the other trees. (For interpretation of the references to colour in this figure legend, the reader is referred to the Web version of this article.)

There were 90 colour images of scenes for the main experiment (60 target scenes that were ‘possible’ which could be in either the semantic or spatial constructive condition, 15 semantic ‘impossible’ scenes, and 15 spatial constructive ‘impossible’ scenes; Fig. 1). The images were matched between conditions in terms of their format (horizontal: 450 pixels (high) x 600 pixels (wide); vertical: 600 × 450 pixels, with fewer pictures being vertical) and whether they were photographs or paintings. Importantly, the content of the images was carefully selected so that each image had semantic and structural elements (e.g., a forest, a building). This selection enabled us to counterbalance the possible images across conditions and participants resulting in two versions of the task. Twelve participants performed test version A (where scene X was in the semantic condition) and 13 participants performed test version B (where scene X was in the spatial constructive condition). To match the impossible images to the possible images, again pictures were chosen with both semantic and structural elements. The impossible images were either taken directly from the internet or images were transformed into impossible images using Adobe Photoshop CS6 (http://www.adobe.com/uk/products/photoshop.html). Pilot testing assured that the difficulty associated with detecting semantic and spatial constructive impossibilities was very similar. The difficulty ratings of the fMRI study participants also endorsed this finding (see Section 3.1).

In addition, there were 30 images (20 possible scenes that could be either semantic or spatial constructive, 5 semantic impossible, and 5 spatial constructive impossible scenes) used for the pre-scan practice, and another 46 images (30 possible, 8 semantic impossible, and 8 spatial constructive impossible scenes) used as lures in the post-scan surprise memory test.

### 2.3 Procedure

Before scanning, participants underwent a short introduction and practice session. They were told that they would be seeing images one at a time and that they had to look carefully at each picture because some of them would depict something that is not really possible. They were given examples of semantically impossible images (e.g., a flying seahorse in a forest, shark fins in a small pond, one of the columns of a hallway made out of books) and spatial constructively impossible images (e.g., trees in a forest or columns of buildings aligned in an impossible manner). For the semantic images, the participants were instructed to check carefully whether the content of a scene looked right to them. For the spatial constructive images, participants were instructed to check carefully whether the spatial elements of a scene appeared to be correct. Participants were then told that a cue (either “content” for semantic images or “structure” for spatial constructive images) would indicate whether there may be a semantic or spatial constructive impossibility in the upcoming scene. In addition, the image itself was surrounded by a green box if participants were to look for semantic and a blue box if they were to look for spatial constructive impossibilities. We took great care to ensure that participants understood the distinction between semantic and spatial constructive impossibilities, since the only difference between our two target conditions (i.e., possible semantic and possible spatial constructive images) was the mental set of the participants. Importantly, we counterbalanced target images (images that had no impossibilities) across participants, so that half of the participants (task version A) looked for semantic and the other half of participants (task version B) looked for spatial constructive impossibilities for a particular possible scene. Following each possible/impossible decision, participants were asked to rate how difficult they found it to decide whether a scene was possible or impossible.

There was also a low-level baseline task that was included in order to allow participants to disengage from the other cognitively challenging tasks. There were 39 such trials, where there was a cross in the centre of the screen that on nine occasions changed its colour from white to red for a brief period to time (500 msec). After each baseline trial, participants were asked whether the colour of the cross had changed and they were instructed to press the first button if the cross had stayed the same colour throughout or the third button if the colour had changed.

Following these instructions, participants completed a practice session on the computer. There were 3 blocks with 9 trials each (i.e., 2 semantic possible, 2 spatial constructive possible, 1 semantic impossible, 1 spatial constructive impossible, 2 baseline, and 1 baseline catch trial). The experiment was run using Cogent 2000 version 125 (Wellcome Centre for Human Neuroimaging, UCL, London, UK). Each trial started with a cue being displayed for 3 s. Next, a scene image was presented for 7.5 s after which the question “Possible?” appeared on the screen (Fig. 1). Participants had then a maximum of 5 s to respond and press the first button on the MRI button box if they thought the current image was possible and the third button if they thought the image was impossible. After participants responded, the difficulty question and its rating scale (1 = not at all, 2 = somewhat, 3 = very) appeared on the screen and participants had again up to 5 s to respond. After each trial of the practice session, the experimenter gave verbal feedback. If there were any mistakes in assigning an image to either possible or impossible, the experimenter brought up the image on the computer screen again after completion of the practice session and explained the difference between both categories for each of the mistakes until the participant fully comprehended the task instructions.

After the practice session, participants were set up in the scanner, and the main experiment began. The timings of the main experiment were identical to the practice session. The main experiment was completed in three blocks of 43 trials (i.e., each block contained 10 semantic possible, 10 spatial constructive possible, 5 semantic impossible, 5 spatial constructive impossible, 10 baseline, and 3 baseline catch trials). The trials were presented in pseudo randomised order so that no more than two images of the same condition were presented consecutively. Completion of the practice and main experiment took each participant approximately 90 min.

### 2.4 Eye-tracking during the fMRI scan

To examine if and how oculomotor behaviour changed depending on the search for either semantic or spatial constructive impossibilities, we recorded eye-tracking data during the main fMRI experiment. We used the MRI compatible Eyelink 1000 Plus (SR Research) eye-tracker. The right eye was used for a 9-point grid calibration, recording and analyses. During the visual search for either semantic or spatial constructive impossibilities, we recorded x and y coordinates of all fixations at a sampling rate of 1000 Hz.

### 2.5 Post-scan surprise memory test

After the scan, participants underwent a surprise memory test. On a computer screen, they saw all 90 scenes from the main experiment again plus 46 lures (30 possible, 8 semantic impossible, and 8 spatial constructive impossible images). In a self-paced manner, participants responded to three questions for each image: 1. Recognition memory: “Do you remember this picture from the earlier scan?” Yes/No; 2. Confidence rating: “How confident are you in your response?” 1 = not at all, 2 = somewhat, and 3 = very much; and 3. Source memory: “If you remember this image from the earlier scan, did you look for a semantic or a structural impossibility?” 1 = semantic, 3 = structural.

Lastly, for the impossible images, we sought to ascertain whether participants identified the correct parts of the images as being impossible. Therefore, we showed participants the images that they rated as impossible again and asked them to indicate with a mouse click on the image where they thought the image was ‘wrong’. All participants performed at ceiling on this test.

### 2.6 Debriefing - strategies

In a debriefing session, we had participants describe what strategies they used to look for either semantic or spatial constructive impossibilities in scenes. We also asked whether participants had seen any of the images before, but none had.

### 2.7 MR image acquisition

All participants underwent functional and structural MRI using a 3 T Magnetom Trio scanner (Siemens Healthcare, Erlangen, Germany). The structural images were collected using a T1-weighted Modified Driven Equilibrium Fourier Transform (MDEFT) sequence with 1 mm isotropic resolution (Deichmann et al., 2004). Functional T2*-weighted images were acquired over three sessions each lasting ~15 min. The sequence was optimised to minimise signal dropout in the vmPFC and medial temporal lobes using a slice tilt of −30° and a z-shim of −0.4 (Weiskopf et al., 2006). The volume TR was 3.36 s, with a TE of 30 msec and echo spacing of 0.5 msec. Per volume, 48 slices were collected in transverse orientation, resulting in a matrix size of 64 × 74 and a 3 mm isotropic voxel size. Following the first functional session, we also acquired a fieldmap with the following parameters: Short TE = 10 msec, Long TE = 12.46 msec, Polarity of phase-encode blips = −1, Applied Jacobian modulation = no, Total EPI readout time = 37 msec, in an ascending slice order.

### 2.8 Data analysis

#### 2.8.1 Behavioural data

Behavioural data collected during the scan and during the post-scan memory test were assessed using separate one-way repeated measures analysis of variance (1way-RM-ANOVA) with experimental condition (i.e., semantic possible, semantic impossible, spatial constructive possible, spatial constructive impossible) as the repeated measure. Where 1way-RM-ANOVAs yielded significant effects (i.e., p < 0.05), we conducted two planned post-hoc comparisons using Sidak's multiple comparison tests – comparing semantic and spatial constructive possible scenes, and comparing semantic and spatial constructive impossible scenes, again considering p-values < 0.05 as statistically significant.

#### 2.8.2 Eye-tracking data

Using the Eyelink Data Viewer (SR Research), we extracted fixation duration, fixation counts, saccade counts and saccade amplitude as dependent measures for each participant for each possible scene image. Given our experimental design, we were able to examine culomotor behaviour for the same scene images with half of the participants searching these images for semantic impossibilities and the other half of participants looking for spatial constructive impossibilities. We therefore calculated for each of the four dependent eye-tracking measures a difference score. For example, those who were administered test version A searched for semantic impossibilities in a particular scene image, while those who experienced test version B searched the same image for spatial constructive impossibilities. We calculated the average fixation counts for version A and version B for each scene, and subtracted the value of version B from that of version A. We then repeated this procedure for every scene image. Therefore, a value around 0 indicated that both test versions elicited similar, for example, fixation counts, a value higher than 0 indicated more fixation counts during the semantic condition, and a value less than 0 indicated more fixation counts during the spatial constructive condition. We used one sample t-tests to assess whether scores diverged from 0, with p < 0.05 considered statistically significant.

#### 2.8.3 MRI pre-processing

All MRI pre-processing was performed using SPM12 (https://www.fil.ion.ucl.ac.uk/spm/). The first five functional images were discarded to allow for signal equilibrium. Functional data were then coregistered to the structural images, realigned and unwarped (including distortion correction with the field maps), normalised to the Montreal Neurological Institute (MNI) template and smoothed with an 8 × 8 X 8 mm kernel FWHM.

#### 2.8.4 fMRI partial least squares (PLS) analysis

A spatiotemporal PLS analysis was performed in order to examine the time course of brain acitivity during semantic and spatial constructive scene processing. PLS is a multivariate, correlational technique that allows for the analyses of associations between brain activity and experimental conditions over each time frame or TR (Krishnan et al., 2011; McIntosh et al., 2004; McIntosh and Lobaugh, 2004). Since PLS does not assume the shape and time course of the hemodynamic response function (HRF), it is important to examine the time duration that would cover the canonical HRF (i.e., around 16 s) despite the presentation of each scene image lasting only 7.5 s. In the current analysis, we therefore analysed 5 consecutive TRs (totalling 16 s).

Detailed descriptions of PLS can be found elsewhere (Krishnan et al., 2011). In brief, PLS uses singular value decomposition (SVD) to extract ranked latent variables (LVs) from the covariance matrix of brain activity and conditions. These LVs express patterns of brain activity associated with each condition, in our case: 1. Possible semantic scenes and 2. Possible spatial constructive scenes. Statistical significance of the LVs was assessed using permutation testing. In this procedure, each participant's data was randomly reassigned (without replacement) to different experimental conditions, and a null distribution was derived from 500 permutated solutions. We considered a LV as significant if p < 0.05. We also assessed the reliability of each voxel that contributed to a specific LV's activity pattern using a bootstrapped estimation of the standard error (i.e., bootstrap ratio, BSR). For each bootstrapped solution (100 in total), participants were sampled randomly with replacement and a new analysis was performed. In the current study, we considered clusters of 20 or more voxels with BSRs greater than 2.5 (approximately equivalent to p < 0.01) to represent reliable patterns of activation.

#### 2.8.5 fMRI seed PSL analysis

As described later in Section 3, the vmPFC was engaged during both semantic and spatial constructive scene processing. We therefore examined vmPFC connectivity using a seed PLS analysis that examines the relationship between a target region (seed voxel) and signal intensities in all other brain voxels as a function of the experimental conditions (Krishnan et al., 2011). The general difference compared to PLS is that in seed PLS the covariance matrix used for SVD stems from correlation values between the seed voxel and all other voxels for each experimental condition (i.e., rather than brain activity values per condition). Using the PLS multivoxel signal extraction toolbox, we extracted signal intensities from the same seed in the vmPFC (peak MNI coordinate −4, 34, −18) separately for the possible semantic and possible spatial constructive scene trials. This seed was selected as it was the peak voxel that shared activation for both types of scene processing. We used a non-rotated version of seed PLS which allowed us to pre-specify a contrast involving vmPFC functional connectivity between semantic and spatial constructive scene processing. Again, we used 500 permutations to assess the significance of a LV, and 100 bootstrap samples to assess reliable voxels. We considered clusters of 20 or more voxels with a BSR greater than 2.5.

#### 2.8.6 fMRI structural equation modelling (SEM)

We next examined the strength of, and directional influences (effective connectivity) among, a set of key areas involved in semantic and spatial constructive scene processing. To do this we used SEM (LISREL 8.80, Student Edition, Scientific Software inc., Mooresville, IN) which examines interregional correlations and anatomical pathways among selected brain areas as the input to compute path coefficients (see McIntosh and Gonzalez-Lima, 1994 for detailed description of SEM for neuroimaging data).

Our region selection for the SEM analyses was based on the highest BSRs and cluster size of the seed PLS analysis as well as functional relevance for semantic processing and scene construction as indicated by the extant literature (e.g., Binder et al., 2009; Summerfield et al., 2010; Zeidman and Maguire, 2016; Zeidman et al., 2015). Ten regions of interest were included in the model (MNI coordinates in brackets): vmPFC (−4, 34, −18), bilateral anterior hippocampi (left = −22, −8, −20; right = 20, −8, −20), bilateral lateral temporal cortices (left = −58, −8, −16; right = 64, −4, −16), bilateral fusiform cortices (left = −24, −78, −10; right = 24, −70, −10), bilateral inferior parietal lobules (left = −48, −62, 48; right = 48, −62, 48), and the right posterior cingulate cortex (10, −50, 34).

An anatomical model of multi-synaptic connections between these regions was derived from known primate neuroanatomy (Catani et al., 2013; Catani et al., 2012; Kondo et al., 2005; Lavenex et al., 2002; Ranganath and Ritchey, 2012; Suzuki and Amaral, 1994). Of note, given our prime interest in the hippocampus and vmPFC, we included connections between the hippocampi and all nodes of the network, as well as between the vmPFC and all nodes of the network. We then constructed functional models for semantic and spatial constructive scene processing. For each participant, the voxel signal intensities were extracted from each chosen region for the semantic and spatial constructive conditions using the PLS multivoxel signal extraction toolbox.

For the path analysis, the correlation matrix of the extracted signal intensities was used to calculate path coefficients, which represented the magnitude of influence of each directional path (McIntosh and Gonzalez-Lima, 1994). We used a stacked-model approach and considered a model improvement of p < 0.05 (Chi-square difference test) as statistically significant.

## 3 Results

### 3.1 In-scanner behavioural performance

Overall, participants performed the possible/impossible task during scanning with high accuracy (overall mean percentage correct 85.6, SD 5.3; see Table 1 for full details of all the in-scanner behavioural measures). While the 1way-RM-ANOVA showed a significant effect of experimental condition (F (3,96) = 22.9, p < 0.0001), the two key planned post-hoc analyses showed no difference in accuracy between the semantic and spatial constructive possible scenes (t = 0.4, df = 96, p = 0.92), or between the semantic and spatial constructive impossible scenes (t = 1.9, df = 96, p = 0.11).

**Table 1:** Summary of task performance.

Scene type	Accuracy		RT (sec)		Difficulty		Recognition		Source		Confidence	
Semantic possible	92.3	*6.2*	1.3	*0.3*	1.7	*0.3*	94.4	*6.7*	65.3	*18.0*	2.8	*0.5*
Semantic impossible	71.7	*11.4*	1.3	*0.3*	1.7	*0.3*	96.2	*6.2*	83.1	*13.3*	2.9	*0.1*
Constructive possible	91.2	*7.1*	1.3	*0.3*	1.8	*0.3*	92.3	*7.4*	69.6	*15.0*	2.9	*0.1*
Constructive impossible	77.6	*15.1*	1.3	*0.3*	1.7	*0.4*	93.4	*7.5*	89.9	*12.6*	2.9	*0.1*

Means and standard deviations (italic) for each scene type are displayed. In-scanner task performance (the three measures shown on the left) included accuracy in percentage of corrected hit rate, RT = reaction times, and difficulty rating for the possible/impossible decisions (1 = not at all … 3 = very much). To the right are the three post-scan measures. Incidental encoding was evaluated with a percentage of corrected hit rate for recognition and source memory, as well as a confidence rating for the recognition memory questions (1 = not at all … 3 = very much).

Analysis of the reaction times for the possible/impossible responses showed no differences between conditions (F (3,96) = 0.2, p = 0.91), and this was also the case for the difficulty ratings (F (3,96) = 0.4, p = 0.73).

### 3.2 Eye-tracking data

**Fig. 2:**
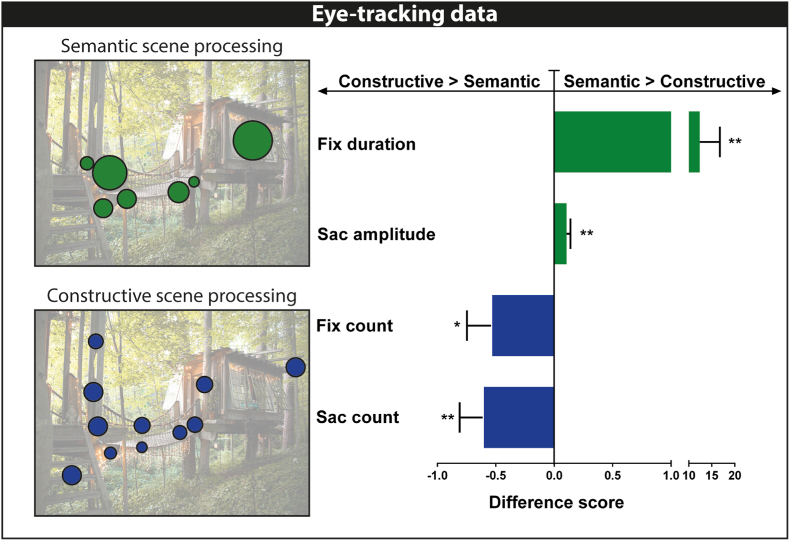
In-scanner oculomotor behaviour. The upper left panel shows eye-tracking data from a representative participant who examined a particular scene for semantic impossibility (green), and the lower left image shows eye-tracking data from a representative participant who examined the same scene for spatial constructive impossibility (blue). The circles indicate fixation positions, and their size indicates fixation durations. The adjacent bar graph displays the results of the analyses of four eye-tracking parameters. * = p < 0.05, ** = p < 0.01. Whereas, on average, participants had more but shorter fixations during spatial constructive than semantic scene processing, they spent longer looking at individual points and then made a greater saccade to a different point on the image during semantic than spatial constructive scene processing. (For interpretation of the references to colour in this figure legend, the reader is referred to the Web version of this article.)

Given that we counterbalanced possible scenes across participants, this allowed us to examine whether oculomotor behaviour differed between the semantic and spatial constructive conditions for the same scenes. We found that participants looked at possible scenes differently depending on whether they were searching for semantic or spatial constructive impossibilities (see Fig. 2). Searching for semantic impossibilities resulted in longer fixations and greater saccade amplitudes compared to searching for spatial constructive impossibilities (fixation duration: t = 2.8, df = 59, p = 0.007; saccade amplitude: t = 3.2, df = 59, p = 0.002). By contrast, when participants looked for spatial constructive impossibilities, fixation and saccade counts were increased compared to searching for semantic impossibilities (fixation count: t = 2.4, df = 59, p = 0.019; saccade count: t = 2.8, df = 59, p = 0.007). These results suggest that participants saccaded more when scrutinising an image for spatial constructive impossibilities, but looked longer at specific parts of an image in order to ascertain whether those parts looked semantically suspicious.

### 3.3 Post-scan behavioural data

After scanning, we administered a surprise memory test. Participants correctly recognised which scenes were presented during scanning and those that were not with high accuracy (overall mean 93 percent, SD 6.9; see Table 1 for full details of the post-scan behavioural measures), and there were no differences between conditions (F (3,92) = 1.2, p = 0.31). Similarly, participants’ confidence ratings were very similar across conditions (F (3,92) = 0.9, p = 0.45).

For each scene that participants recognised from the main experiment, they were asked to indicate whether they had looked for semantic or spatial constructive impossibilities (i.e., source memory). Examining the corrected hit rate revealed a significant effect (F (3,92) = 14.4, p < 0.0001). However, the two key planned post-hoc analyses showed no difference in source memory between the semantic and spatial constructive possible scenes (t = 1.0, df = 24, p = 0.54), or between the semantic and spatial constructive impossible scenes (t = 1.6, df = 24, p = 0.21).

We asked participants to describe their strategies for searching scenes for semantic or spatial constructive impossibilities. They reported approaches that aligned with the eye-tracking data. For the semantic condition, 22 out of 25 participants described that they looked briefly at a scene but then spent most of their time focussing on individual elements of a scene and considering whether they fitted well or whether there was anything unusual. For example, typical responses of participants for the semantic task were: “Did the scene make sense? I tried to think abstractly”; “I thought about semantic associations”; “I searched the image. Was there something odd?”. For the spatial constructive condition, 20 out of 25 participants reported that they scanned the spatial layout, as well as closely scrutinising the structure, perspective, angles, and intersections within the scenes. They also described building an internal model of the scene to help judge whether the structure deviated from what would be expected. Here, typical responses for the spatial constructive task included: “I checked the construction, compared lines where you would expect them to end up”; “I looked at the perspective of the whole image”; “I examined the space and compared it to how it would look like in real life”.

### 3.4 fMRI PLS

**Fig. 3:**
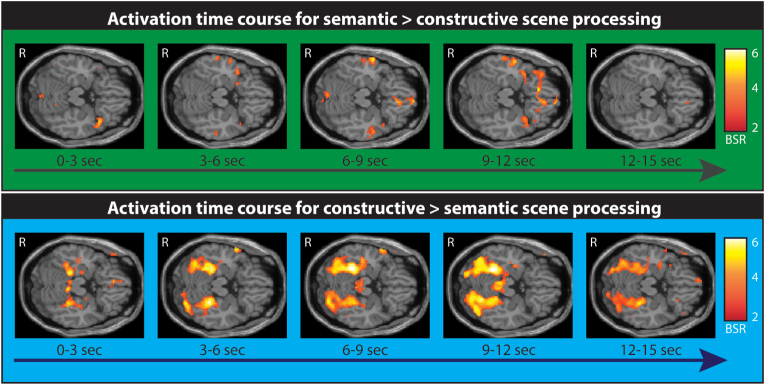
Temporal characteristics of semantic and spatial constructive scene processing as revealed by an fMRI PLS analysis. The upper panel shows the increased activations for semantic versus spatial constructive scene processing across five time windows exceeding a bootstrap ratio (BSR) of 2.5 and a cluster size of >20 contiguous voxels. The vmPFC and lateral temporal cortices were engaged at a later time window (6–9 s). The lower panel shows the increased activation for spatial constructive versus semantic scene processing. Of note, the vmPFC and bilateral anterior hippocampi were engaged very early on within the first time window (0–3 s). Activations are displayed on a T1-weighted MRI (MNI template), R = right, sec = seconds.

The spatiotemporal PLS analysis revealed a significant LV that differentiated between semantic and spatial constructive scene processing (p < 0.006; Fig. 3; see Tables S1 and S2 for details of all the brain regions that were engaged). This LV showed that the brain areas involved and their temporal activation characteristics differed between conditions. Unpacking these results first for semantic scene processing, we found increased activity in the lateral temporal cortices, lateral and medial parietal cortices, and the vmPFC. By contrast, spatial constructive scene processing was associated with engagement of regions that included bilateral anterior medial hippocampi, vmPFC, and several areas along the ventral visual stream. Of note, activation in both hippocampi and the vmPFC peaked together, at a very early time point. The remaining time windows mostly showed sustained activation in visual-perceptual areas. Interestingly, some of the vmPFC voxels that showed co-activation early on with bilateral hippocampi during the spatial constructive condition, were also engaged at a later time point during the semantic condition but now co-activated with bilateral lateral temporal cortices. Therefore, we next examined the functional connectivity of the peak vmPFC voxel that the two conditions shared in common.

### 3.5 fMRI seed PLS

**Fig. 4:**
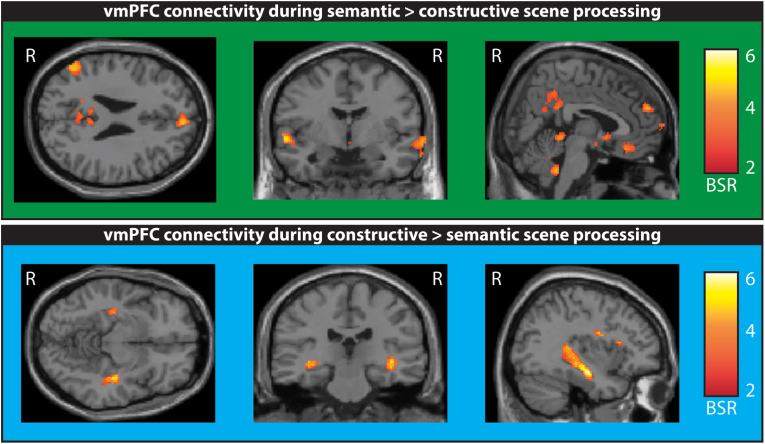
Functional connectivity of the vmPFC during semantic and spatial constructive scene processing as revealed by a seed PLS analysis. The upper panel shows increased functional vmPFC connectivity for semantic versus spatial constructive scene processing exceeding a bootstrap ratio (BSR) of 2.5 and a cluster size of >20 contiguous voxels. The vmPFC was connected to lateral temporal cortices, and lateral and medial parietal cortices. The lower panel illustrates increased functional vmPFC connectivity for spatial constructive versus semantic scene processing. The vmPFC was strongly connected to the entire length of both hippocampi. Functional connectivity is displayed on a T1-weighted MRI (MNI template), R = right.

We found a significant pattern that separated the two conditions (p < 0.004; Fig. 4; see Table S3 for details of all correlated brain regions). During semantic scene processing the vmPFC was most strongly connected to bilateral lateral temporal cortices and lateral and medial parietal cortices. By contrast, during spatial constructive scene processing the vmPFC was functionally connected to both hippocampi over their entire length.

### 3.6 fMRI SEM

**Fig. 5:**
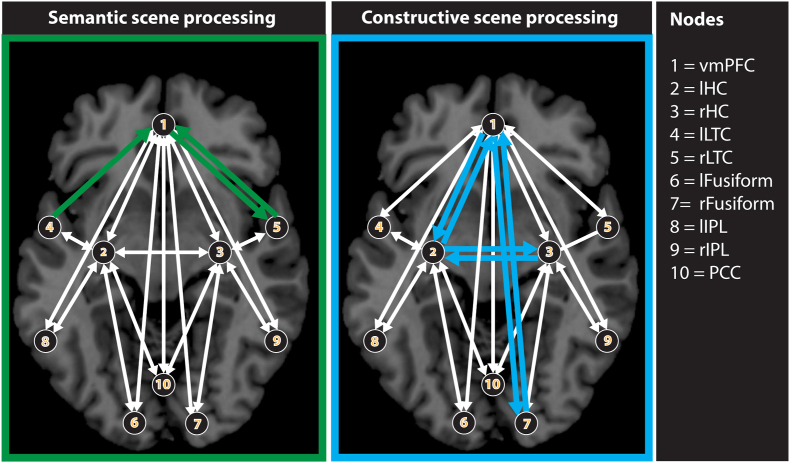
Effective connectivity during semantic and spatial constructive scene processing. The left panel shows the effective connections that were stronger during semantic than spatial constructive scene processing (green arrows), and the right panel the effective connections that were stronger during spatial constructive than semantic scene processing (blue arrows). White arrows represent anatomical connections that were included in the model but did not differ between the two conditions. Of note, we included connections between vmPFC and all other nodes in the model as well as connections between the hippocampi and all other nodes. The vmPFC interacted with distinct task-specific areas during semantic (lateral temporal cortices) and spatial constructive (hippocampi and visual-perceptual cortices) scene processing. VmPFC = ventromedial prefrontal cortex, lHC = left hippocampus, rHC = right hippocampus, lLTC = left lateral temporal cortex, rLTC = right lateral temporal cortex, lFusiform = left fusiform gyrus, rFusiform = right fusiform gyrus, lIPL = left inferior parietal lobule, rIPL = right inferior parietal lobule, PCC = posterior cingulate cortex. (For interpretation of the references to colour in this figure legend, the reader is referred to the Web version of this article.)

The previous seed-based approach showed the functional connectivity changes for one particular seed region. The multivariate SEM effective connectivity analyses we conducted next had the added advantage of examining connectivity changes across various regions. We examined effective connectivity within a set of areas comprising the vmPFC, anterior hippocampi, lateral temporal cortices, fusiform gyrus, and lateral and medial parietal cortices (Fig. 5; see Tables S1 and S2 for all regions and their connections that were included in the SEM analysis). We found that the model in which path coefficients were free to vary was a better fit for our data than the model in which path coefficients were fixed (CHIdiff = 119.37, df = 36, p < 0.001, all stability indices ≤1). This indicated that directional connectivity differed significantly between semantic and spatial constructive scene processing.

We found nine connections that differed significantly between our conditions (path coefficients are given in brackets). During semantic scene processing, the vmPFC was strongly connected to the lateral temporal cortices (left LTC to vmPFC, semantic: 0.4, spatial constructive: 0.1; right LTC to vmPFC, semantic: 0.4, spatial constructive: −0.2; vmPFC to right LTC, semantic: 0.2, spatial constructive: −0.1). Of note, hippocampal connectivity did not feature during semantic scene processing. During spatial constructive scene processing, there were strong recurrent interactions between the vmPFC and left hippocampus (vmPFC to left hippocampus, semantic: −0.4, spatial constructive: 0.3; left hippocampus to vmPFC, semantic: −0.4; spatial constructive: 0.4), and between the vmPFC and right fusiform gyrus (vmPFC to right fusiform gyrus, semantic: −0.5; spatial constructive: 0.5; right fusiform gyrus to vmPFC, semantic: −0.1, spatial constructive: 0.1). There was also recurrent interactions between both hippocampi (left to right, semantic: −0.1, spatial constructive: 0.3; right to left, semantic: 0.4; spatial constructive: 0.6).

## 4 Discussion

Scene imagery features prominently when we recall autobiographical memories, imagine the future and navigate around in the world (Clark et al., 2020). Consequently, in this study we sought to better understand how scene representations are supported by the brain. Processing scenes involves a variety of cognitive processes that in the real world are highly interactive. Here, however, our goal was to separate semantic and spatial constructive scene processes to identify the brain areas that were distinct to each process, those they had in common, and the connectivity between regions. Participants searched for either semantic or spatial constructive impossibilities in scenes, and we focussed our analyses on only those scenes that were possible, thus removing any error detection processes that evoked reactions such as surprise, novelty or ‘a-ha’ responses. Importantly, we counterbalanced possible scenes across participants, enabling us to examine brain activity and connectivity for the same possible scene images under two different conditions. Our fMRI findings, therefore, could not be stimulus driven, nor was there any difference in incidental encoding. Instead, the participants adopted different cognitive strategies, which were reflected in distinct oculomotor behaviour, for each condition. These were in turn associated with increased engagement of lateral temporal and parietal cortices for semantic scene processing, the hippocampus for spatial constructive scene processing, with increased activation of the vmPFC common to both. Connectivity analyses showed that the vmPFC switched between semantic and spatial constructive brain networks depending on the task at hand.

When participants focussed on semantic aspects of scenes this was accompanied by increased activity in several brain regions relative to spatial constructive scene processing, including lateral temporal cortices, medial and lateral parietal cortices, as well as frontal cortices. This finding aligns closely with findings from a large meta-analysis of neuroimaging studies that examined the semantic system in healthy individuals (Binder et al., 2009; see also Chadwick et al., 2016; McAndrews et al., 2016; Silveira et al., 2012). Moreover, lateral temporal regions are frequently damaged in conditions such as herpes simplex encephalitis (Warrington and Shallice, 1984; Kapur et al., 1994; Gitelman et al., 2001; Lambon Ralph et al., 2007; Noppeney et al., 2007) and semantic dementia (Warrington, 1975; Snowden et al., 1989; Hodges et al., 1992, 1995; Mummery et al., 2000; Jefferies and Lambon Ralph, 2006; Lambon Ralph et al., 2007; Noppeney et al., 2007), often resulting in profound semantic deficits. Binder et al. (2009) proposed that the category-related deficits in these patients, and fMRI studies more generally, point to the temporal lobe being a principal site for storage of perceptual information about objects and their attributes.

In line with this idea, we found longer fixation durations and larger saccade amplitudes when participants assessed semantic scene content compared to the spatial constructive condition. In fact, similar patterns of eye-movements have been associated previously with the allocation of attention to objects and their semantic relationship with the scene context in which the objects appeared (Einhaeuser et al., 2008; Loftus and Mackworth, 1978; Spotorno and Tatler, 2017). The majority of our participants reported that their general strategy was to look briefly at a scene but then spent most of their time focussing on individual elements of a scene and considering whether they fitted well or whether there was anything unusual. Interestingly, the fMRI activation time course may have reflected this strategy, showing as it did a slower temporal evolution for semantic compared to spatial constructive scene processing. This included the peak of the co-activation of the vmPFC and lateral temporal cortices falling within the third and fourth time window.

By contrast, and in agreement with previous studies, we found stronger activation along the ventral visual stream, as well as bilateral anterior medial hippocampi during spatial constructive scene processing compared to semantic scene processing (Hassabis et al., 2007b; Summerfield et al., 2010; Zeidman et al., 2015; Aly et al., 2013; Ruiz et al., 2020). This finding also accords with previous studies that showed that the hippocampi and surrounding medial temporal lobes are involved in detecting spatial constructive impossibilities in scenes (Douglas et al., 2017; McCormick et al., 2017a). For example, patients with focal bilateral hippocampal damage could detect semantic impossibilities in scenes but were impaired at identifying spatial constructive impossibilities (McCormick et al., 2017a; but see Urgolites et al., 2019). Of note, these previous neuropsychological and fMRI studies involved participants spotting the impossibility, and so engaged error detection processes that evoked reactions such as surprise and novelty. In contrast, our analysis focussed on possible scenes, and so we can more easily relate our findings to spatial constructive processing rather than to these other factors.

Interestingly, the peak of hippocampal (and vmPFC) engagement fell into the first time window of the fMRI analysis. This fast response mirrors those recently observed in several MEG studies. For instance, Monk et al. (2021) examined the step-by-step construction of scene imagery and found modulation of theta power in the anterior hippocampus and vmPFC during the earliest stage of construction. Similarly, McCormick et al. (2020) found that the hippocampus and vmPFC were engaged very early during autobiographical memory retrieval. This early neural engagement for spatial constructive processes has parallels with the subjective reports of our participants, where they described that searching for spatial constructive impossibilities relied upon an instantaneous scanning of the image for structural coherence. Moreover, the distinct oculomotor behaviour associated with spatial constructive scene processing involved shorter fixations and more saccades but with smaller amplitudes, when compared to semantic scene processing. These findings also support the suggested link between hippocampal involvement and fast visual sampling (El Haj and Lenoble, 2018; Hannula and Ranganath, 2009; Liu et al., 2017; Voss et al., 2017). Of note, our data speak against the idea that the hippocampus necessarily guides viewing patterns based on memory (Voss et al., 2017). In our task, the memory demand was identical across conditions, yet we observed different eye movement patterns for identical scenes depending on the task demands. Hence, it may be that the process rather than the memory load per se predicts hippocampal involvement, and our findings support the view that the hippocampus-related process in question may be spatial constructive (Hassabis and Maguire, 2007; Maguire and Mullally, 2013; Zeidman and Maguire, 2016). This interpretation aligns with recent perspectives focussing on the relationship between hippocampal engagement and fixation count as an indicator of visual imagery and mental event construction (Ryan et al., 2020; Conti and Irish, 2021).

In this study the vmPFC was active during both semantic and spatial constructive scene processing. This observation supports the view that the vmPFC may play a hierarchically superordinate role in scene processing (McCormick et al., 2018; Ciaramelli et al., 2019). The vmPFC was co-activated early on with bilateral hippocampi, and later on with bilateral lateral temporal cortices, findings that we investigated further with functional and effective connectivity analyses. We found that the same region of the vmPFC was functionally connected to the lateral temporal as well as lateral and medial parietal cortices during semantic scene processing, and to the hippocampus during spatial constructive scene processing. Transient network dynamics during complex cognitive tasks, such as autobiographical memory retrieval involving the vmPFC and hippocampus, have been observed previously (McCormick et al., 2015, 2017b; St Jacques et al., 2011). In fact, a vmPFC region close to the current seed location switched transient functional connectivity from anterior to posterior segments of the hippocampi during an early and late stage of autobiographical memory recall (McCormick et al., 2017b).

Of note, whereas these earlier studies mostly examined hippocampal-centred networks, in our SEM analyses we included anatomical connections between the vmPFC and all other regions, as well as between the hippocampus and all other regions. Thus, we were able to differentiate between a vmPFC- and a hippocampal-centred network model. Interestingly, we found that the vmPFC had specialised effective interactions with task-specific brain areas, such as lateral temporal cortices during semantic scene processing and hippocampus and visual-perceptual cortices during spatial constructive scene processing. By contrast, the hippocampi were mainly connected to one another and to the vmPFC. Consequently, our data suggest that the vmPFC (rather than the hippocampus), with its access to both semantic and spatial constructive information, may be best positioned to orchestrate scene processing. Nevertheless, it remains an open question as to whether the vmPFC or the other brain areas drive these connectivity changes. Our SEM revealed mainly recurrent interactions. However, recent MEG studies showed that the vmPFC was the driving influence during scene construction (Monk et al., 2020) and autobiographical memory recall (McCormick et al., 2020). It would be interesting to conduct the paradigm here in MEG to elucidate the temporal dynamics and effective connectivity further.

Also regarding future studies, given our finding of vmPFC involvement in both semantic and spatial constructive scene processing, it would be useful to revisit the testing of patients with vmPFC damage. De Luca et al. (2019) found no differences between the patients and control participants in detecting either semantic or spatial constructive violations using the McCormick et al. (2017a) task. However, as noted previously, the nature of that task may have offered a means to circumvent performance issues. Perhaps a different task can be devised involving fewer specific cues to test the necessity of vmPFC involvement in scene processing further.

In conclusion, the paradigm we used here allowed us to explore transient brain networks associated with processing different aspects, semantic and spatial constructive, of well-matched scenes. The findings further highlight the well-known semantic functions of lateral temporal areas (Binder et al., 2009), while providing additional support for the previously-asserted contribution of the hippocampus to scene construction (Hassabis and Maguire, 2007; Maguire and Mullally, 2013), and recent suggestions that the vmPFC may play a key orchestrating role in scene processing (McCormick et al., 2018; Ciaramelli et al., 2019).
