# Supplementary material for: The distinct and overlapping brain networks supporting semantic and spatial constructive scene processing
Source: Neuropsychologia. 2021 Jul 30;158:107912. doi: 10.1016/j.neuropsychologia.2021.107912 (PMC8287593; doi:10.1016/j.neuropsychologia.2021.107912)
Supplement: Multimedia component 1 [file mmc1.docx]

**The distinct and overlapping brain networks supporting semantic and spatial constructive scene processing**

**Cornelia McCormick, Eleanor A. Maguire**

Supplementary Tables

Table S1: Peak coordinates of the contrast semantic > spatial constructive scene processing

Table S2: Peak coordinates of the contrast spatial constructive > semantic scene processing

Table S3: Peak coordinates of vmPFC connectivity during scene processing

**Table S1: Peak coordinates of the contrast semantic > spatial constructive scene processing**

|  |  |  |  |  |  |  |  |
| --- | --- | --- | --- | --- | --- | --- | --- |
|  | **Hemisphere** | **X** | **Y** | **Z** | **BSR** | **Size** | **SEM** |
| **Lag 1** |  |  |  |  |  |  |  |
| Inferior frontal gyrus | R | 22 | 30 | 16 | 5.23 | 399 |  |
| Inferior frontal gyrus | L | -58 | 22 | 2 | 4.75 | 652 |  |
| Superior frontal gyrus | R | 24 | 50 | 0 | 4.66 | 322 |  |
| Cingulum gyrus | R | 14 | -22 | 40 | 4.25 | 153 |  |
| Superior temporal pole | R | 62 | 10 | -4 | 3.61 | 86 |  |
| Inferior parietal lobule | L | -40 | -50 | 42 | 3.59 | 429 |  |
| Middle frontal gyrus | L | -46 | 12 | 52 | 3.44 | 333 |  |
| Supramarginal gyrus | L | -64 | -20 | 16 | 3.41 | 99 |  |
| Middle frontal gyrus | R | 50 | 2 | 54 | 3.33 | 45 |  |
| Inferior parietal lobule | R | 42 | -48 | 44 | 3.28 | 180 |  |
| Middle temporal gyrus | L | -48 | -46 | 8 | 3.08 | 722 |  |
| Precuneus | L | -8 | -68 | 46 | 2.95 | 33 |  |
| Cerebellum | R | 28 | -86 | -32 | 2.86 | 25 |  |
| Superior temporal gyrus | R | 68 | -30 | 18 | 2.71 | 71 |  |
| Middle temporal gyrus | R | 64 | -52 | 10 | 2.63 | 32 |  |
| Inferior temporal gyrus | L | -40 | -10 | 0 | 2.58 | 20 |  |
| Middle frontal gyrus | L | -36 | 34 | 40 | 2.53 | 30 |  |
| Middle frontal gyrus | R | 34 | 54 | -8 | 2.51 | 24 |  |
| Supramarginal gyrus | R | 62 | -40 | 38 | 2.49 | 43 |  |
| Precuneus | R | 12 | -64 | 42 | 2.45 | 49 |  |
| Middle frontal gyrus | R | 38 | 16 | 60 | 2.41 | 41 |  |
|  |  |  |  |  |  |  |  |
| **Lag 2** |  |  |  |  |  |  |  |
| Inferior parietal lobule | L | -38 | -58 | 42 | 4.99 | 1784 |  |
| Precuneus | L | -8 | -48 | 38 | 4.75 | 632 |  |
| Middle frontal gyrus | L | -42 | 14 | 52 | 4.57 | 1103 |  |
| Anterior cingulate gyrus | R | 20 | 42 | -2 | 4.33 | 556 |  |
| Cerebellum | L | -2 | -48 | 0 | 3.69 | 119 |  |
| Inferior frontal gyrus | L | -46 | 30 | -2 | 3.68 | 94 |  |
| Caudate | L | -18 | 0 | 22 | 3.65 | 73 |  |
| Medial frontal gyrus | L | -20 | 52 | 0 | 3.17 | 403 |  |
| Cerebellum | R | 28 | -86 | -34 | 3.15 | 154 |  |
| Middle temporal gyrus | R | 66 | -28 | -14 | 3.12 | 37 |  |
| Middle temporal gyrus | R | 70 | -30 | 4 | 3.06 | 70 |  |
| Middle frontal gyrus | L | -28 | 36 | 20 | 2.98 | 98 |  |
| Medial frontal gyrus | R | 12 | 54 | 20 | 2.93 | 60 |  |
| Superior temporal pole | R | 40 | 8 | -20 | 2.87 | 101 |  |
| Inferior frontal gyrus | L | -52 | 14 | 0 | 2.76 | 41 |  |
| Inferior parietal lobule | R | 42 | -64 | 44 | 2.66 | 214 |  |
|  |  |  |  |  |  |  |  |
| **Lag 3** |  |  |  |  |  |  |  |
| **Inferior parietal lobule** | **L** | **-48** | **-62** | **48** | **4.62** | **1209** | ***** |
| **Inferior parietal lobule** | **R** | **48** | **-62** | **48** | **4.55** | **430** | ***** |
| **Posterior cingulate gyrus** | **R** | **10** | **-50** | **34** | **4.00** | **856** | ***** |
| Cerebellum | R | 26 | -78 | -32 | 3.58 | 345 |  |
| **Middle temporal gyrus** | **R** | **64** | **-4** | **-16** | **3.44** | **78** | ***** |
| Middle frontal gyrus | L | -24 | 26 | 40 | 3.41 | 719 |  |
| Medial frontal gyrus | L | -6 | 62 | -14 | 3.36 | 375 |  |
| **Medial frontal gyrus (vmPFC)** | **L** | **-4** | **34** | **-18** | **3.21** | **90** | ***** |
| Superior frontal gyrus | L | -18 | 58 | 10 | 3.14 | 372 |  |
| Inferior frontal gyrus | L | -46 | 22 | 20 | 2.95 | 56 |  |
| Inferior frontal gyrus | L | -46 | 30 | -2 | 2.89 | 94 |  |
| **Middle temporal gyrus** | **L** | **-58** | **-8** | **-16** | **2.81** | **153** | ***** |
| Superior temporal gyrus | L | -58 | -26 | -2 | 2.77 | 242 |  |
| Superior frontal gyrus | L | -16 | 36 | 42 | 2.76 | 107 |  |
| Cerebellum | L | -24 | -82 | -38 | 2.44 | 36 |  |
|  |  |  |  |  |  |  |  |
| **Lag 4** |  |  |  |  |  |  |  |
| Medial frontal gyrus | L | -6 | 60 | -12 | 4.21 | 300 |  |
| Medial frontal gyrus | R | 6 | 26 | -8 | 3.90 | 183 |  |
| Caudate | R | 12 | 4 | 26 | 3.60 | 54 |  |
| Cerebellum | R | 16 | -88 | -32 | 3.14 | 214 |  |
| Inferior parietal lobule | R | 52 | -56 | 50 | 3.03 | 23 |  |
| Superior temporal gyrus | R | 34 | 12 | -22 | 2.98 | 116 |  |
| Middle temporal gyrus | R | 64 | -6 | -20 | 2.88 | 87 |  |
| Supramarginal gyrus | L | -60 | -56 | 30 | 2.87 | 31 |  |
| Inferior frontal gyrus | R | 42 | -32 | 22 | 2.79 | 20 |  |
| Superior frontal gyrus | R | 14 | 50 | 0 | 2.70 | 31 |  |
| Superior frontal gyrus | L | -14 | 50 | 42 | 2.67 | 62 |  |
| Superior temporal gyrus | R | 58 | -60 | 24 | 2.65 | 37 |  |
| Medial frontal gyrus | L | -10 | 40 | -16 | 2.65 | 60 |  |
| Superior temporal gyrus | L | -46 | -64 | 50 | 2.57 | 35 |  |
| Superior temporal pole | L | -38 | 10 | -20 | 2.53 | 46 |  |
|  |  |  |  |  |  |  |  |
| **Lag 5** |  |  |  |  |  |  |  |
| Middle frontal gyrus | L | -24 | 38 | -10 | 3.52 | 298 |  |
| Posterior cingulate gyrus | R | 10 | -42 | 14 | 3.43 | 198 |  |
| Anterior cingulate gyrus | R | 4 | 22 | -4 | 3.16 | 121 |  |
| Insula | R | 30 | -14 | 22 | 2.93 | 29 |  |
| Insula | R | 44 | -6 | 18 | 2.84 | 34 |  |
| Medial frontal gyrus | L | -6 | 62 | 8 | 2.67 | 34 |  |
| Cerebellum | R | 28 | -80 | -32 | 2.60 | 43 |  |
| Superior temporal pole | R | 42 | 18 | -32 | 2.46 | 26 |  |

X,Y, and Z coordinates in MNI space, BSR=Bootstrap ratio, Size=Cluster size in voxels, R=right, L=left, SEM=Structural Equation Modelling, *=Regions included in the SEM analysis

**Table S2: Peak coordinates of the contrast spatial constructive > semantic scene processing**

|  |  |  |  |  |  |  |  |
| --- | --- | --- | --- | --- | --- | --- | --- |
|  | **Hemisphere** | **X** | **Y** | **Z** | **BSR** | **Size** | **SEM** |
| **Lag 1** |  |  |  |  |  |  |  |
| Parahippocampal gyrus | L | -18 | -40 | -12 | -5.79 | 959 |  |
| **Fusiform gyrus** | **R** | **24** | **-70** | **-10** | **-4.24** | **430** | ***** |
| **Fusiform gyrus** | **L** | **-24** | **-78** | **-10** | **-3.74** | **337** | ***** |
| Middle occipital gyrus | L | -38 | -76 | 24 | -2.99 | 318 |  |
| Postcentral gyrus | R | 52 | -24 | 44 | -2.97 | 96 |  |
| Inferior frontal gyrus | R | 64 | 22 | 16 | -3.89 | 87 |  |
| Cerebellum | R | 6 | -52 | -34 | -2.88 | 64 |  |
| Middle temporal gyrus | L | -50 | -58 | -6 | -2.65 | 57 |  |
| Superior occipital gyrus | R | 28 | -86 | 34 | -2.74 | 56 |  |
| Middle frontal gyrus | R | 4 | 26 | -12 | -2.92 | 55 |  |
| Superior parietal lobule | R | 24 | -70 | 50 | -2.60 | 51 |  |
| **Medial frontal gyrus (vmPFC)** | **L** | **-4** | **34** | **-18** | **-2.87** | **96** | ***** |
| Inferior frontal gyrus | R | 60 | 40 | -2 | -2.77 | 45 |  |
| Precuneus | L | -14 | -78 | 52 | -2.43 | 45 |  |
| Superior parietal lobule | R | 26 | -54 | 60 | -3.01 | 43 |  |
| Lingual gyrus | R | 18 | -74 | -12 | -3.07 | 42 |  |
| **Hippocampus** | **L** | **-22** | **-8** | **20** | **-2.67** | **35** | ***** |
| **Hippocampus** | **R** | **20** | **-8** | **-20** | **-2.66** | **33** | ***** |
| Thalamus | L | -12 | -26 | -2 | -2.66 | 20 |  |
|  |  |  |  |  |  |  |  |
| **Lag 2** |  |  |  |  |  |  |  |
| Superior frontal gyrus | R | 22 | 2 | 56 | -8.29 | 1937 |  |
| Fusiform gyrus | R | 26 | -62 | -12 | -7.43 | 6872 |  |
| Inferior parietal lobule | R | 36 | -38 | 48 | -7.42 | 12088 |  |
| Parahippocampal gyrus | L | -30 | -40 | -8 | -7.09 | 4324 |  |
| Middle frontal gyrus | L | -24 | 2 | 56 | -6.25 | 1040 |  |
| Inferior frontal gyrus | R | 52 | 12 | 26 | -5.16 | 873 |  |
| Middle temporal gyrus | R | 62 | 6 | -24 | -4.80 | 83 |  |
| Cerebellum | L | -12 | -44 | -28 | -4.20 | 761 |  |
| Precentral gyrus | L | -50 | 4 | 26 | -3.69 | 294 |  |
| Insula | L | -34 | -4 | 12 | -3.31 | 48 |  |
| Insula | R | 40 | -2 | 4 | -3.20 | 61 |  |
| Inferior frontal gyrus | R | 36 | 34 | 16 | -3.14 | 256 |  |
| Superior temporal gyrus | L | -30 | 16 | -28 | -3.12 | 39 |  |
| Putamen | R | 30 | -14 | -6 | -2.91 | 38 |  |
|  |  |  |  |  |  |  |  |
| **Lag 3** |  |  |  |  |  |  |  |
| Supramarginal gyrus | R | 38 | -36 | 46 | -7.90 | 31200 |  |
| Superior frontal gyrus | R | 26 | 0 | 52 | -6.92 | 1970 |  |
| Fusiform gyrus | R | 24 | -70 | -10 | -6.73 | 6872 |  |
| Parahippocampal gyrus | L | -34 | -40 | -6 | -6.49 | 4324 |  |
| Superior frontal gyrus | L | -22 | -2 | 44 | -6.29 | 1047 |  |
| Inferior frontal gyrus | R | 54 | 12 | 20 | -5.03 | 1191 |  |
| Inferior frontal gyrus | L | -50 | 6 | 30 | -4.48 | 355 |  |
| Inferior frontal gyrus | R | 56 | 44 | 0 | -4.14 | 208 |  |
| Inferior frontal gyrus | R | 24 | 28 | -8 | -4.09 | 304 |  |
| Middle frontal gyrus | R | 44 | 44 | 16 | -3.55 | 616 |  |
| Superior temporal pole | R | 64 | 12 | -12 | -3.27 | 78 |  |
| Insula | R | 42 | -2 | 2 | -2.93 | 85 |  |
|  |  |  |  |  |  |  |  |
| **Lag 4** |  |  |  |  |  |  |  |
| Parahippocampal gyrus | R | 34 | -48 | -4 | -7.20 | 40324 |  |
| Cerebellum | L | -12 | -54 | -46 | -5.60 | 1663 |  |
| Cerebellum | R | 12 | -52 | -48 | -3.68 | 56 |  |
| Middle frontal gyrus | L | -54 | 48 | -4 | -3.60 | 43 |  |
| Cuneus | R | 6 | -88 | 34 | -3.33 | 32 |  |
| Cingulate gyrus | R | 18 | 24 | 30 | -3.06 | 33 |  |
| Postcentral gyrus | L | -14 | -36 | 62 | -2.70 | 31 |  |
| Insula | L | -30 | 16 | 4 | -2.63 | 126 |  |
| Insula | L | -34 | 22 | 26 | -2.55 | 63 |  |
|  |  |  |  |  |  |  |  |
| **Lag 5** |  |  |  |  |  |  |  |
| Superior parietal lobule | R | 22 | -74 | 54 | -5.18 | 14341 |  |
| Inferior frontal gyrus | R | 56 | 44 | 0 | -3.97 | 1608 |  |
| Cerebellum | L | -16 | -50 | -44 | -3.72 | 392 |  |
| Middle frontal gyrus | L | -56 | 44 | -4 | -3.19 | 113 |  |
| Anterior cingulate gyrus | R | 4 | 2 | 28 | -3.16 | 79 |  |
| Precuneus | L | -12 | -50 | 18 | -2.86 | 88 |  |
| Middle occipital gyrus | L | -16 | -94 | 6 | -2.78 | 90 |  |
| Inferior frontal gyrus | L | -38 | 4 | 28 | -2.75 | 109 |  |
| Superior frontal gyrus | R | 20 | 0 | 60 | -2.68 | 73 |  |
| Caudate | L | -20 | -20 | 22 | -2.68 | 21 |  |
| Superior frontal gyrus | R | 20 | 62 | -8 | -2.67 | 25 |  |
| Superior frontal gyrus | L | -10 | -62 | -34 | -2.66 | 90 |  |
| Cerebellum | L | -32 | -60 | -42 | -2.53 | 29 |  |

X,Y, and Z coordinates in MNI space, BSR=Bootstrap ratio, Size=Cluster size in voxels, R=right, L=left, SEM=Structural Equation Modelling, *=Regions included in the SEM analysis

**Table S3: Peak coordinates of vmPFC connectivity during scene processing**

|  |  |  |  |  |  |  |
| --- | --- | --- | --- | --- | --- | --- |
|  | **Hemisphere** | **X** | **Y** | **Z** | **BSR** | **Size** |
| **Semantic > Constructive** |  |  |  |  |  |  |
| Superior temporal gyrus | R | 52 | -60 | 20 | 7.19 | 417 |
| Medial frontal gyrus | R | 4 | 30 | -12 | 5.71 | 401 |
| Cerebellum | L | -52 | -58 | -26 | 5.45 | 142 |
| Medial frontal gyrus | R | 18 | 64 | 8 | 5.07 | 1339 |
| Middle temporal gyrus | L | -58 | -4 | -4 | 5.03 | 266 |
| Superior temporal gyrus | R | 68 | -2 | -6 | 4.82 | 148 |
| Superior frontal gyrus | L | -6 | 54 | 28 | 4.61 | 115 |
| Cerebellum | R | 52 | -62 | -30 | 4.31 | 163 |
| Precuneus | L | -8 | -46 | 46 | 4.07 | 196 |
| Superior frontal gyrus | L | -12 | 34 | 60 | 3.94 | 63 |
| Caudate | L | -10 | 14 | 4 | 3.85 | 184 |
| Supplementary Motor Area | R | 2 | 10 | 70 | 3.77 | 65 |
| Precuneus | R | 8 | -54 | 16 | 3.74 | 83 |
| Putamen | R | 24 | 18 | 2 | 3.69 | 70 |
| Inferior parietal lobule | L | -45 | -50 | 44 | 3.52 | 306 |
| Putamen | L | -22 | 12 | -10 | 3.40 | 56 |
| Posterior cingulate gyrus | R | 4 | -42 | 28 | 3.33 | 115 |
|  |  |  |  |  |  |  |
| **Constructive > Semantic** |  |  |  |  |  |  |
| Medial frontal gyrus | R | 2 | 28 | -12 | -9.14 | 1670 |
| Inferior temporal gyrus | L | -38 | -8 | -30 | -7.16 | 1127 |
| Cerebellum | R | 28 | -66 | -40 | -6.43 | 891 |
| Posterior cingulate gyrus | L | -4 | -40 | 30 | -6.27 | 358 |
| Fusiform gyrus | R | 30 | -32 | -20 | -5.78 | 141 |
| Insula | R | 34 | 20 | 10 | -5.37 | 116 |
| Superior temporal pole | R | 62 | 16 | -12 | -5.34 | 485 |
| Inferior frontal gyrus | R | 56 | 44 | 2 | -5.07 | 54 |
| Superior frontal gyrus | L | -2 | 68 | 22 | -5.06 | 1411 |
| Superior temporal pole | R | 66 | -32 | 16 | -4.59 | 553 |
| Middle frontal gyrus | L | -54 | 24 | 30 | -4.53 | 52 |
| Middle occipital gyrus | R | 52 | -66 | -8 | -4.44 | 210 |
| Cingulate gyrus | L | -12 | -12 | 40 | -4.44 | 473 |
| Precuneus | R | 10 | -68 | 42 | -4.33 | 335 |
| Postcentral gyrus | R | 22 | -34 | 78 | -4.33 | 100 |
| Cerebellum | L | -40 | -74 | -34 | -4.31 | 324 |
| Posterior cingulate gyrus | R | 12 | -42 | 26 | -4.29 | 64 |
| Globus pallidus | L | -24 | -10 | -4 | -4.01 | 66 |
| Anterior cingulate gyrus | R | 12 | 44 | 18 | -3.98 | 63 |
| Medial frontal gyrus | R | 2 | 62 | -8 | -3.91 | 191 |
| Precuneus | L | -2 | -64 | 18 | -3.74 | 108 |
| Superior frontal gyrus | R | 16 | -10 | 76 | -3.69 | 303 |
| Posterior cingulate gyrus | R | 12 | -40 | 12 | -3.65 | 77 |
| Hippocampus | L | -20 | -14 | -20 | -3.60 | 95 |
| Hippocampus | R | 20 | -10 | -14 | -3.58 | 45 |
| Middle temporal gyrus | L | -62 | -60 | 2 | -3.40 | 95 |
| Fusiform gyrus | R | 42 | -8 | -30 | -3.27 | 57 |
|  |  |  |  |  |  |  |

X,Y, Z coordinates in MNI space; BSR=Bootstrap ratio; Size=Cluster size in voxels; R=right; L=left
